# Social media usage among health care providers

**DOI:** 10.1186/s13104-017-2993-y

**Published:** 2017-11-29

**Authors:** Zoya Surani, Rahim Hirani, Anita Elias, Lauren Quisenberry, Joseph Varon, Sara Surani, Salim Surani

**Affiliations:** 1 Veterans Memorial High School, Corpus Christi, TX USA; 2 School of Natural Science, Hampshire College, Amherst, MA USA; 3 Sanamed Education, Miami, FL USA; 4 Texas Tech University, El Paso, TX USA; 5 The University of Texas Health Science Center at Houston, Houston, TX USA; 6 The University of Texas, Medical Branch at Galveston, Houston, TX USA; 7 Harvard University, Cambridge, MA USA; 8 Texas A&M University, Corpus Christi, TX USA; 9 University of North Texas, 1177 West Wheeler Ave, Aransas Pass, TX 78366 USA

**Keywords:** Social media, Healthcare workers, Physicians, Nurses, Depression, Policy

## Abstract

**Objective:**

The objective of this study was to evaluate the use of social media among healthcare workers in an attempt to identify how it affects the quality of patient care.

**Results:**

An anonymous survey of 35 questions was conducted in South Texas, on 366 healthcare workers. Of the 97% of people who reported owning electronic devices, 87.9% indicated that they used social media. These healthcare workers indicated that they spent approximately 1 h on social media every day. The healthcare workers below the age of 40 were more involved in social media compared to those above 40 (*p* < 0.05). The use of social media among physicians and nurses was noted to be identical (88% for each group), and both groups encouraged their patients to research their clinical conditions on social media (*p* < 0.05). A higher number of physicians reported awareness of a social media policy in their hospital compared to nurses (*p* < 0.05). However, a large proportion of healthcare workers (40%) were unaware of their workplace policy, which could potentially cause a privacy breach of confidential medical information. Further studies are required to evaluate specific effects of these findings on the quality of patient care.

## Introduction

Seventy percent of Americans use social media and connect with each other, currently, which can be compared to only 5% of Americans, which used social media for this purpose in 2005 [1]. Social media can be considered one of the most innovative and one of the most destructive market forces which has emerged. Health care is not immune to it either. Currently, more than 40% of the health care consumers utilize social media for their healthcare information needs. This being even more in the consumers who are 18–24 years age group, compared to the 45–54 year age group. 90% of the health care consumers in the 18–24 year age group utilizes and believe health care information presented in the social media [2].

Whether through advertising or interactions with other colleagues, hospitals and healthcare workers engage with one another via social media in their work fields [3]. 41% of the health care consumers chose social media to decide on their health care providers. In addition 26% of the hospitals in USA is currently participating in the social media in some form [2]. Social media has provided to be a communication system for healthcare workers through which they can find patient health information and past medical history or they can review relevant information about illnesses [4]. In terms of benefits, social media has provided healthcare providers a way to overcome barriers in delivering healthcare to patients [5], has enhanced self-management skills among patients who can familiarize themselves with specific illnesses and also provides numerous opportunities for providers to conduct research in their relevant fields [6]. Some clinicians have suggested that social media is among the best ways to design, collect, and analyze data into scientific papers for academic journals [7].

However, potential harmful outcomes can result from social media and should be considered, especially as they relate to patient confidentiality [8]. Many hospitals and practices have strict social media policies, but not all employees are aware of them.

Additionally, age is thought to be directly correlated with the time spent on social media [9]. One study concluded that medical students, who are younger, spend more time on social media as compared to the residents, which suggests this correlation [10]. Another study showed that the use of social media among physicians was 41% in 2010 and 90% in 2011, as compared to medical students who usage was more than 90% [3]. Thus taking into account the factor of age could help us narrow down the general age group to work with in order to reduce the harmful effects of using social media during working hours, as it could be the source of distraction. Moreover, only 31% of healthcare institutions have social media policy, per Institute of Health [2].

The purpose of our study was to understand healthcare workers knowledge regarding the social media policies in their institution; their utilization of social media and also the utilization of healthcare providers above and below the age of 40 years of age, as the data has shown the utilization of social media is significantly higher in populations below the age of 45 years.

## Main text

### Study design

This study was an anonymous exploratory research using sampling of convenience. It analyze the most commonly used social media, estimate daily time spent on social media between health care providers, analyze subject awareness regarding their hospital’s social media policy, and to see whether healthcare workers encouraged their patients to use it as a tool to educate themselves regarding disease process. An Ethics and Institutional Review Board (IRB) of Corpus Christi Medical Center, Corpus Christi Texas USA approved the survey and protocols. Consent waiver was granted by the IRB. The survey was carried out in a local community hospital and specialty clinic of mid-size suburban town.

All health care providers in the coastal bend area of Texas can be included. The healthcare providers who do not desire to fill the complete questionnaire or unable to read the questionnaires were excluded.

### Methods

A sample size of 360 was needed based on the population of the health care providers in the coastal bend region of Texas to be 6000. A total of 366/370 healthcare provides participated (Table 1). Since there was not a validated questionnaire available, and being a pilot study, the questionnaire, comprised of 35 questions was developed, after deliberating the questionnaire among the investigators. These questions were pretested among five physicians and five nurses. These were modified again based on the feedback from this pilot group. These questionnaires were anonymously distributed and collected in the healthcare setting. The responses from the paper questionnaire were manually entered and analyzed electronically, mainly using Microsoft Excel, Microsoft Corporation, California USA. p values were calculated using two-tail Student *t* test. p value of < 0.05 was considered significant.

**Table 1:** Demographics of participants

Category	Subcategory	N (%)
Gender	Male	108 (29.5)
	Female	257 (70.2)
	No response	1 (0.3)
Race	African American/Black/Caribbean	12 (3.3)
	Asian/Pacific Islander	40 (11.0)
	Caucasian	148 (40.8)
	Hispanic/Latino	156 (43.0)
	Native American	2 (0.6)
	Other	5 (1.4)
Age group	< 20	0 (0)
	20–29	92 (25.3)
	30–39	123 (33.8)
	40–49	64 (17.6)
	50 +	85 (23.4)
Job	Physician	68 (19.0)
	RN	151 (42.3)
	LVN	10 (2.8)
	CMA	6 (1.7)
	PA	2 (0.6)
	Dietitian	1 (0.3)
	Speech pathologist	1 (0.3)
	Pharmacist	5 (1.4)
	Pharmacy technician	5 (1.4)
	Physical therapist	7 (2.0)
	Other	101 (28.3)

*RN* registered nurses, *LVN* license vocational nurses, *CMA* certified medical assistants, *PA* physician assistants

### Results

366 surveys were analyzed. 108 participants were male (29.5%), 257 were female (70.2%), and 1 preferred not to respond (0.3%) (Table 1). Hispanics (43.0%) and Caucasians (40.8%) dominated the sample size (Table 1). 97% of the sample size indicated that they own a type of electronic device. Of those 97%, 87.9% of indicated that they used some kind of social media on a regular.

Out of 366 participants, 217 were under the age of 40, and 151 participants were over 40 (Table 1). In the under-40 group, 91.2% of participants used social media compared to 84.1% in the over 40 group. In < 40 age group, 78.3% of participants used social media > 30 min per day compared to 62.3% of participants in > 40 year age group. In > 40 year age group, 85.3% of participants and 68.2% of participants in < 40 year age group have been involved in social media for more than a year. Our study also revealed that 71.7% of participants in the < 40 and 42.7% in the > 40 age group use social media before going to bed. The statistical comparison on the use of social media between these two age groups is shown in Table 2.

**Table 2:** Statistical comparison on the use of social media between two age groups (< 40 and > 40 years)

#	Item description	< 40 years of age (n = 217) (%)	> 40 years of age (n = 151) (%)	*p* values
1	Do you use social media?	91.2	84.1	0.036*
2	More or less than 30 min?	78.3	62.3	0.00070*
3	Involvement in social media (< 1 year or > 1 year)	85.3	68.2	0.00008*
4	Do you think you waste your time on social media?	31.3	33.8	0.62
5	Do you use social media before going to bed?	71.7	42.7	0.000001*
6	Do you think social media takes away from your family time?	14.4	20.8	0.11
7	Do you get less sleep because of using social media?	16.6	15	0.68

* *p* values < 0.05

35.4% of healthcare providers indicated that they spent 31–60 min/day, 32.0% selected < 30 min/day, 16.8% selected 61–90 min/day, 8.1% selected 91–120 min/day, and 7.8% selected > 2 h/day.

Among daily users of social media, 76.5% of the participants spent < 10% of the time on social media for work related activities, whereas 1.7% indicated their use was mostly work related. 32.3% of the participants indicated that their use of social media is a waste of time.

As per their institutional social media policy, 40.8% of subjects were unaware of the policy’s existence.

Of the physicians and nurses, 66% indicated that patients could access their medical records online, and 42.8% encouraged their patients to become familiar with their maladies by reading about them online.

Differences in the use of social media were found between the physicians (n = 69) and nurses (n = 152) in the sample population (Table 1). The use of social media among physicians and nurses was observed to be identical at 88% for each group. While 10.4% of physicians contributed to medical forums online, only 3.3% of nurses indicated the same. Information available online regarding disease processes was considered reliable by 37.9% of physicians compared to 47.5% of nurses, and 61.5% of physicians and 56.8% of nurses encourage their patients to search about their illnesses and diseases processes online. Awareness of the hospital’s social media policies was reported in 72.2% of physicians compared to 38.2% of nurses. A statistical comparison is shown in Table 3.

**Table 3:** Statistical comparison on the use of social media between physicians and nurses

#	Item description	RN (n = 152) (%)	MD (n = 69) (%)	p values
1	Use of social media	88	88	> 0.05
2	Do you contribute to medical forums online?	3.3	10.4	0.03*
3	Do you think information on social media is correct?	47.5	37.9	0.53
4	Do you recommend your patients to search about their illness online?	56.8	61.5	0.02*
5	Are you aware of your social media policy?	72.2	38.2	0.000008*

* *p* values < 0.05

This study indicated that even though less than 50% of physicians and nurses believed that online information on disease processes was reliable, both groups encouraged their patients to pursue it (Table 3).

### Discussion

Social media, and the internet in general, is expanding rapidly all over the world [11, 12]. In our study, 87.9% of the participants indicated the use of social media, which is similar to prior studies [13]. The use of social media in healthcare settings is increasing daily as it pertains to community engagement, promotion of health, patient education, outreach, and various other factors. The consumers are now driving the increase usage [14, 15].

To provide protection for the privacy of patients’ medical records, a law was enacted by the United States Congress in 1996 known as The Health Insurance Portability and Accountability Act (HIPAA) (Centers for Disease Control and Prevention (CDC) [16]). The consequences in the breach of privacy can be severe and may lead to civil or criminal penalties [17].

Social media can also be a large distraction in the workplace. With all the perks of accessing medical records easily through different websites or applications, it is also highly tempting to use that resource for recreational purposes during work [18]. One study showed that when Facebook™ use was allowed in the workplace, productivity decreased by 1.47% [18]. In another study, institutions that allowed their participants to use Facebook™ every day for 15 min demonstrated a decrease in work efficiency by 1.5% [19].

Our study suggests that social media is an important tool for healthcare providers to help familiarize patients with their clinical conditions. This is especially beneficial for those who are unable to access healthcare information easily, including ethnic minorities and lower socioeconomic groups [20]. In one study, 53.5% of the patients who participated, indicated the use of social media for medical and health information [21]. However, social media may be deleterious for patient care.

To our knowledge, there are no previous studies quantifying the number of healthcare providers recommending the use of social media to their patients. We found that nearly half of participants (42.8%) encouraged their patients to read about disease processes on social media.

Our study suggested that a large number of healthcare workers are unaware of the social media policy at their workplace. This is worrisome because understanding institutional policies regarding social media is crucial in protecting confidential medical information and avoiding HIPPA compliance issues. Moreover, if healthcare providers are unaware of their social media policy, this can affect medical professionalism as a whole. It is important to note that though unintentional, this example is a violation of HIPAA, which restricts an individual from posting medical images to protect patient privacy [22]. Even after organizations such as the AMA provided these guidelines, not many healthcare providers are aware of or in compliance with their social media policies [23]. In one study, only 54% of healthcare institutions surveyed had a social networking policy [24]. Our result is consistent with these data (59.2%).

Some studies have shown that the use of social media decreases with age [25]. In one survey, 83% of the participants who were active users on social media were under the age of 30 years [25]. In our study, healthcare workers below the age of 40 used more social media and spent considerably longer amounts of time on it compared to healthcare workers above the age of 40 (*p* < 0.05). These differences are multifactorial. In our study, more healthcare workers who were above the age of 40 indicated that social media takes away from their family time.

### Conclusion

The use of social media in our cohort was in line with the general public. However, our study suggests that physicians contribute to medical forums online more than nurses. More than half of physicians and nurses encouraged their patients to learn about their disease processes online, however, only a small percentage believe that the information regarding the illness is reliable, which in contrast to what the consumers believe. Further studies are needed to determine the impact of social media on patterns of healthcare quality and disparities of care.

## Limitations

Small sample size, encompassing single community hospital and clinic limits it results to be applied to the larger population. Moreover, cultural and geographical differences among population across the world may limit the generalization of this paper and hence similar studies in multiple geographical locations are required. Study design being sampling of convenience, also places some limitations. Not having a validated questionnaire also is a limitation of this study. We though suggest development of validated questionnaire and conducting a randomized trial to address this important issue which health care industry is facing.

## Abbreviations

USA: United States of America
IRB: Institutional Review Board
CDC: Center for Disease Control and Prevention
